# Reply to Cooper and Stanojevic: ‘Is lung function in a race against time?’

**DOI:** 10.1113/EP093614

**Published:** 2026-02-15

**Authors:** Samuel Wallbanks

**Affiliations:** ^1^ Birmingham Heartlands Hospital University Hospitals Birmingham NHS Foundation Trust Birmingham UK

## 1 INTRODUCTION

I read with interest the editorial by Professor Brendan Cooper and Professor Sanja Stanojevic, ‘Is lung function in a race against tim?’ (Cooper & Stanojevic, 2024). Their piece offers a thoughtful and necessary reflection on the historical origins of race‐based adjustments in lung function reference equations, and the damaging legacy these practices have left within medicine. The editorial argues persuasively that the historical misuse of race to differentiate lung function values has contributed to entrenched inequities in respiratory healthcare, and that moving towards race‐neutral equations (Bowerman et al., 2023) rather than Global Lung Initiative 2012 values (Quanjer et al., 2012) may represent an important opportunity to ‘level up’ fairness and reduce bias.

I want to begin by stating clearly that I fully acknowledge, and do not dispute, the racist history associated with lung function equations and the unethical misuse of physiology to justify social hierarchies (Braun, 2015). My opposition to aspects of race‐neutral reference equations relate to the following: (i) Are we ready to ignore ancestry‐related physiological variation in reference values? (ii) Do race‐neutral equations genuinely reduce health inequities – or do they risk creating new ones? and (iii) Does implementation threaten workflow continuity, clinical safety and diagnostic robustness?

## 2 ARE WE READY TO IGNORE ANCESTRY‐RELATED PHYSIOLOGICAL VARIATION IN REFERENCE VALUES?

For clarity, *race* is used to describe groups with shared physical characteristics (e.g., skin colour), while *ethnicity* refers more broadly to cultural, social and ancestral commonalities. Normative reference equations aim to allow clinicians to compare an individual's results to a population with shared biological characteristics. For lung function, most of the variation is explained by age, height and sex but the rest is explained by factors we do not understand, one of which is race and the environments in which people live and grow.

It is agreed that more genetic variation exists within, rather than between, ethnic groups listed within the original GLI 2012 race‐specific values. However, if race‐by‐environmental interactions had little explanatory power on lung function, the choice of specific ethnic categories within GLI 2012 would have minimal clinical impact. In practice, this is certainly not the case. It is acknowledged that racial categorisations are more overlapping than separate (Yudell & Hammonds, 2024); however, it is unclear whether excluding the smaller proportion of shared physiological commonalities risks discarding clinically meaningful information.

There is some biological plausibility for race‐based differences in respiratory function and disease. As an example, telomere length is naturally longer in people with darker skin, notably in those with African ancestry. Shorter telomere length is a genetic risk factor for progressive interstitial lung disease and alters the processes of age‐related cell senescence in both the skin and the lung tissues (Adegunsoye et al., 2023). Race has been acknowledged as an important factor in the disease behaviours within interstitial lung disease as well as other respiratory diseases (Adegunsoye et al., 2018). Morphological differences also exist: with individuals ancestrally closer to the equator having longer limb length relative to torso size as an adaptation to support evaporative heat loss. This, in turn, modulates lung function. Are we not to acknowledge that racial differences in bodily proportions exist?

These observations do not justify race as a biological proxy (Yudell & Hammonds, 2024). However, they do raise the question of whether a ‘one‐size‐fits‐all’ approach risks oversimplifying a complex physiological reality. The only true way to reliably measure what is normal lung function for a given individual is to perform spirometry once the person has reached maximally attained lung function between the ages of 20 and 30 years, and measure normality longitudinally from this value. Given the complexity of influences on lung function and the range of factors not fully understood, it seems incongruous to ignore the impact of race. It is agreed that there are flaws in how race is reported and documented (Braun, 2015). However, ignoring this removes clinically meaningful information.

## 3 DO RACE‐NEUTRAL EQUATIONS GENUINELY REDUCE HEALTH INEQUITIES – OR DO THEY RISK CREATING NEW ONES?

Within lung function reference values, there is a trade‐off between data heterogeneity and clinical usefulness of reference values. Greater inclusion of heterogenous lung function data widens the ranges of normal, which which leads to more false negative results in the context of significant disease. The range of normal was already too wide with GLI 2012 values, with as much as 2 litres between the upper and lower limit of normal in certain age groups (Wallbanks & Apen, 2024). The race neutral values extend this further, due to the increased heterogeneity of analysing Caucasian, Black American, North and Southeast Asian individuals collectively. The wider range of normal with race neutral values directly contrast public health priorities, where respiratory diseases are more under‐ than over‐diagnosed (Bradley et al., 2023). Race neutral values, although socially conscious, may inadvertently cause harm and impede early detection of respiratory disease.

The effects of race neutral values will also not be distributed evenly. Individuals previously classified as Caucasian in GLI 2012 may experience up to 15% predicted increases in measured lung function values under race‐neutral equations, pseudo‐normalising their values (Rosenfeld et al., 2024). Others, previously categorised as Black American, will experience a reduction in their predicted reference values, quite rightly, increasing the chances of detection. Whilst this will certainly increase equitability, it has to be asked, is equitability desirable if it means jeopardising the diagnostic sensitivity of spirometry for Caucasians? Whether the approach is truly ‘race neutral’ is therefore contested.

## 4 DOES IMPLEMENTATION THREATEN WORKFLOW CONTINUITY, CLINICAL SAFETY AND DIAGNOSTIC ROBUSTNESS?

A further concern is the impact of new reference values on clinical pathways. Clinical workflows in respiratory medicine are tightly aligned to lung function reference values. These values guide referral decisions for lung transplantation, lung volume reduction, access to clinical trials, initiation of specific therapies, and even eligibility for social support systems. Sudden recalibration of predicted values shifts all these thresholds.

Such disruption risks diagnostic delay, misclassification and increased clinician uncertainty. In interstitial lung disease, spirometry is already often ‘towards the lower ends of normal’ in the context of significant radiological fibrosis and symptomology. Particularly for Caucasians, race‐neutral values will require system re‐design and greater reliance on clinical correlation of spirometry – a factor that will lead to more medical errors in the context of health systems under pressure.

An additional unintended consequence concerns data recording. If race‐neutral equations remove the need to document race or ethnicity, fewer spirometry reports may record this. A lot of research on health inequity by race relies on describing outcomes of these groups retrospectively. If race and ethnicity disappear from the dataset, so too may the ability to study disparities.

A final point is that the race‐neutral equations are more based on statistics rather than increasing the inclusivity of those included in the equations. The race‐neutral equations utilise the same dataset as GLI 2012 but aggregate all the populations. Notably, a range of groups were omitted from the initial GLI 2012 equations due to excess heterogeneity, particularly for Oman, Iran and Pakistan where sample sizes were actually large (Quanjer et al., 2012). A key question is why is it now acceptable to increase the heterogeneity of lung function reference values in the race neutral equations? And if we now collate heterogenous groups together and statistically analyse collectively without adding representation of marginalised groups, have we actually improved equity? Or have we simply redistributed the same bias, now hidden beneath the brand of race neutrality?

## AUTHOR CONTRIBUTIONS

Sole author.

## CONFLICT OF INTEREST

The author declares he has no conflicts of interest.

## FUNDING INFORMATION

No funding was received for this work.
